# Perceived benefits of smoke-free homes, the process of establishing them, and enforcement challenges in Shanghai, China: a qualitative study

**DOI:** 10.1186/s12889-015-1428-8

**Published:** 2015-02-06

**Authors:** Carla J Berg, Pinpin Zheng, Michelle C Kegler

**Affiliations:** Department of Behavioral Sciences and Health Education, Emory University School of Public Health, 1518 Clifton Road, NE, Room 524, Atlanta, GA 30322 USA; Key Laboratory of Public Health Safety, Ministry of Education, School of Public Health, Fudan University, 138 Yixueyuan Road, Shanghai, 200032 China

**Keywords:** Smoking, Tobacco control, Secondhand smoke, Health communications

## Abstract

**Background:**

We examined reasons for establishing smoke-free home policies, interpersonal processes by which they are established, and challenges in enforcing them in Shanghai, China.

**Methods:**

In 2013, we conducted 30 in-person semi-structured interviews among 13 male smokers and 17 female nonsmokers recruited from urban and a suburban communities in Shanghai.

**Results:**

Reasons for adopting a smoke-free home included family’s health, being a role model for children, cleaner environment, and potential impact on smoking behavior. Wives were credited with initiating discussion regarding the implementation of a smoke-free home most often and were reported to have decision-making authority. Some households had not discussed such a rule. Common responses to asking to establish a smoke-free home among husbands were agreeing not to smoke at home or in front of family members, ignoring the request, temporarily acquiescing, insisting on smoking in the home anyway, and devaluing the benefits of smoke-free homes. Challenges to enforcement included weather, social situations, the smoker being home alone, ineffective harm reduction behaviors such as smoking near windows, and addiction were challenges in enforcement.

**Conclusions:**

Specific factors (e.g. family’s health) could be highlighted to assist women, men, and children in adopting and enforcing smoke-free home policies.

## Background

With a population of 1.2 billion, China is the world’s largest producer and consumer of tobacco. The high prevalence of current (past 30 day) smoking among adults (men: 52.9%; women: 2.4%) in China [1] equates to about with over 350 million smokers [2]. As a result of the ratification of the Framework Convention on Tobacco Control (FCTC) in August 2005, significant changes in the national tobacco control policy have been made by the Chinese Government [3,4]. As such, current national and local efforts focus on expanding smoke-free environments by restricting smoking in schools, hospitals, worksites, or public places such as bars or restaurants; many of these environments have limited or no restrictions on smoking [5]. With the extensive efforts to establish smoke-free workplaces and public venues globally, in many countries the home is becoming the predominant source of exposure to second-hand smoke (SHS) among children and other non-smokers in the household even in countries without advanced public smoke-free policies [5,6].

Very little is known about the smoking behavior of Chinese smokers when they are in the home, particularly with respect to exposing children and other non-smoking household members to SHS. The World Health Organization (WHO) estimates that nearly half of the world’s children (roughly 700 million) are exposed to SHS at home [7]. The high prevalence of adult male smoking and the fact that there are few smoking restrictions in place in China suggest that a large number of young children are exposed to SHS in Chinese homes. In the 2008–2010 Global Adult Tobacco Survey in China, the prevalence of SHS exposure among non-smokers (aged 15 or above) was 64.1% among men and 63.2% among women. The Global Youth Tobacco Survey conducted in 2005 in Shanghai indicated that, among primary school students, three-quarters of children had one or more parents who smoked and nearly half (47.0%) lived in homes where others in the household smoked in their presence [8]. This is a true public health risk given that children’s exposure to SHS is high and that children may have little influence on household decisions regarding smoking indoors.

Some prior research has examined reasons for establishing smoke-free homes in China. One qualitative study in China [9] found that, although there was a lack of knowledge about the health risk of exposure to SHS, most adults were willing to protect their children from SHS exposure. Moreover, our prior research has found that health concern was the most important reason for establishing a smoke-free home in homes in Shanghai, followed by “other” concerns (e.g., avoid annoying others with SHS, avoiding house fires) and concerns about cleanliness [10]. Participants with complete smoke-free home policies reported greater health concerns, children-related concerns, cleanliness concerns, and other concerns, compared to those without a smoke-free policy [10].

Little research has examined the interpersonal interactions within households in China that lead to the implementation of smoke-free home policies. Our recent research examined correlates of having a smoke-free home in Shanghai among 500 participants in urban and suburban districts, 35.3% of whom had a complete smoke-free home policy [10]. In addition to being a nonsmoker and having higher income, important factors associated with having a smoke-free home were social factors, specifically not having smokers in the home, having children in the home, and having fewer friends/relatives who permit smoking at home. This indicates that social interactions among household members regarding the establishment of a policy may be important in this process. Prior research has found that many families do not openly discuss smoking or smoking restrictions at home and that barriers to adopting a smoke-free home included the social acceptability of smoking, hosting social gatherings at home or having visitors, and authoritative attitudes of the husband or father-in-law [9,11]. In addition, individuals with no or a partial ban commonly allow smoking in the living room, the kitchen, and the bathroom, all of which are common areas of the home, underscoring the challenges of making the communal areas in the home smoke-free [10].

There are several gaps in the literature regarding perceived benefits or motivators for going smoke-free, the process of establishing them, and how they are enforced, particularly in low- and middle-income countries. As such, the aims of this study were to examine smoking practices and rules in the home, perceived benefits of or motivators for implementing a smoke-free home policy, interactions leading to implementing smoke-free homes, whom had authority to establish the smoke-free home, husbands’ reactions to home smoking rules, and challenges or exceptions regarding enforcement in Shanghai, China. This data will provide useful baseline information on smoke-free homes and perhaps allow more effective SHS exposure reduction programs to be developed for Chinese families.

## Methods

### Participants and procedures

In Spring 2013, we conducted 30 in-person semi-structured interviews to explore our study aims. Semi-structured interviews are well-suited to explore individual subjective experiences and attitudes, particularly related to concepts or phenomenon that have not been well explored previously [12]. Thus, this qualitative approach was selected due to the limited prior research related to perceptions of secondhand smoke, smoke-free home policies, and barriers to and facilitators of adopting and enforcing smoke-free home policies. The Institutional Review Boards of Emory University and Fudan University approved this study.

We recruited participants from an urban and a suburban community in Shanghai. In each community, we posted fliers in one kindergarten, one primary school, and one secondary school. We intentionally screened for participants living with at least one child and fitting each of the following criteria: male current (past 30-day) smokers living with at least one nonsmoker with partial or no smoke-free home policy; male current smokers living with at least one nonsmoker with complete smoke-free policies; female nonsmokers living with at least one smoker with partial or no smoke-free home policy; and female nonsmokers living with at least one smoker with a complete smoke-free home policy. This sampling frame was employed in order to ensure representation across these different groups and to be able to examine any possible nuances in responses among the groups. We initially planned to recruit at least 10 male smokers and 10 female nonsmokers and at least 10 participants with complete smoke-free policies and 10 without in order to ensure sufficient numbers in these groups. We recruited additional participants subsequently to ensure that we had reach saturation. Interested individuals called the research team at Fudan University, were screened for eligibility, were informed about the study, and were scheduled for an in-person interview. Each participant was compensated with 100 RMB for participation.

Interviews were conducted in conference rooms on campus (n = 27) or in the homes of participants (if participants could not come to campus; n = 3). Prior to the interview, participants completed the informed statement of consent indicating that participation was completely voluntary and that they were free to withdraw from the study at any time. Three interviewers (the second author and two trained Masters of Public Health [MPH] level staff) guided the interviews, which lasted approximately 40 minutes. Once saturation was reached within a category of participants, recruiting participants for that category was discontinued [12].

### Measures

Measures for the current study included a semi-structured interview guide adapted from a similar study within the U.S. [13] with a brief structured section assessing sociodemographic and smoking-related history. Each of the measures was translated from English to Chinese by bilingual MPH students at Emory and Fudan and then back-translated into English by bilingual MPH students at Emory and Fudan to ensure that meaning was consistent across languages.

#### Structured assessment

We assessed sociodemographics (age, gender, ethnicity, marital status, monthly household income, education level, type of occupation); type of housing (single unit/detached home; townhome/duplex; apartment/condo/multi-unit complex); smoking history (whether the participant had ever smoked 100 cigarettes in his or her lifetime, the number of days smoked of the past 30 days, and the number of cigarettes smoked per day [CPD] among current [past 30 day] smokers); household composition (i.e., number of people and smokers in the home, number of children under the age of 18); and rules about smoking in cars and homes with response options of “no rules”, “smoking allowed in some places or sometimes”, or “smoking is never allowed” (for cars, “no car”).

#### Semi-structured interview guide

The interview covered the following topics: 1) smoking practices and rules in the home, 2) perceived benefits or motivators for implementing a smoke-free home policy, 3) interactions leading to implementing smoke-free homes, 4) whom had authority to establish the smoke-free home, 5) husbands’ reactions to home smoking rules, and 6) challenges or exceptions regarding enforcement.

### Data analysis

Descriptive statistics from the structured interview were computed using means and standard deviations for continuous variables and frequency and percentage for categorical variables. Quantitative data were analyzed using SPSS 21.0 (IBM Corporation, Armonk, NY).

All sessions were audio-taped and transcribed. Chinese transcripts were independently reviewed by the interviewers to ensure accuracy, and then were translated into English by two bilingual MPH students at Fudan. The English transcripts were compared to the Chinese transcripts by two bilingual MPH students at Emory to ensure consistency of information and meaning.

Then, the second author and two research project staff members, both of whom were bilingual Master of Public Health students trained in qualitative analyses, generated preliminary codes using an inductive and deductive process. The research team used an iterative process to develop a master coding structure [14,15]. Primary (i.e., major topics explored) and secondary codes (i.e., recurrent themes within these topics) were then clearly defined in a codebook that was used to independently code each transcript. Qualitative data was coded and organized using NVivo 10.0 (QSR International, Cambridge, MA). Thematic content analysis was conducted by two independent coders to identify themes, and matrices were constructed to help identify patterns and themes by gender. Coders resolved any discrepancies through discussions. Intra-class correlations for context exceeded 0.96. Themes were then identified and agreed upon between the second author and the coders, and representative quotes were selected.

## Results

Of the 30 participants, 43.3% (n = 13) were male smokers (5 with a complete smoke-free policy; 8 without a complete policy), and 56.7% (n = 17) were female nonsmokers (9 with a complete smoke-free policy; 8 without a complete policy; see Table 1). The majority were between the ages of 30 and 49 (90.0%; n = 27), had a college education or greater (53.3%; n = 16), and lived in an apartment (86.7%; n = 26). Among the male smokers, they smoked an average of 26.9 (SD = 6.2) days in the past 30 days and an average of 11.1 (SD = 6.2) CPD on smoking days. Table 2 provides the themes that emerged for each of the topics discussed and sample quotations representing each theme. Any differences among men and women and among those with or without complete smoke-free home policies are noted below.

**Table 1:** **Sociodemographics and smoking characteristics of study participants**

**Characteristics**	**Total**
	**N = 30**
*Sociodemographics*	
Gender/smoking status	
Male current smoker	13 (43.3)
Female nonsmoker	17 (56.7)
Ethnic	
Han	29 (96.7)
Other	1 (3.3)
Age	
20-29	2 (6.7)
30-39	16 (53.3)
40-49	11 (36.7)
50-59	1 (3.3)
Education	
Less than high school	5 (16.7)
High school graduated	9 (30.0)
College or higher	16 (53.3)
Monthly household income	
Less than 3000 Yuan	7 (23.3)
3001-5999 Yuan	12 (40.0)
6000-7999 Yuan	7 (23.3)
More than 8000 Yuan	4 (13.3)
Marital status	
Married	30 (100.0)
Other	0 (0.0)
Residence	
Apartment	26 (86.7)
Single house	4 (13.3)
Number of people in the home	3.8 (0.9)
Number of children in the home	1.1 (0.3)
Smoking characteristics*	
Days smoked, past 30 days	26.9 (6.2)
CPD on smoking days	11.1 (6.2)

*Among current smokers.

**Table 2:** **Themes and sample quotations regarding smoking practices and rules in the home, perceived benefits or motivators for implementing a smoke-free home policy, interactions leading to implementing smoke-free homes, and enforcement challenges in Shanghai, China**

**Theme**	**Sample quotation**
***Smoking practices and rules in the home***	
*Locations where smoking is allowed*	“Smoking is only allowed in the living room and balcony. In fact, smoking occurs most frequently in the living room, because we watch television and have guests come to visit sit there. It is also a place to rest. It has larger space, so the air circulates faster than other places.” (FA01)
	“It is because there is a range hood in the kitchen that can take the smoke out. If smoking is allowed in the bedroom or the living room, there will be much smoke smell and residue remains.” (FA03)
	“We stay in the bedroom longer. In addition, it is comparatively closed off with less air circulation. If someone smokes there, the smell will remain for a long time and I cannot stand it. My children are still young.” (FA08)
	“Generally, he does not smoke in son’s presence. Therefore, his smoking won’t affect the child.” (FA05)
*Triggers for smoking*	“It’s hard to stop him smoking after having meals, because smokers are used to smoking after meals.” (FA04)
	“He may smoke one or two cigarettes in the computer room while play computer.” (FF03)
	“When he is playing mahjong or watching TV, he husband smokes frequently.” (FA02)
	“When he is working at home, sometimes writing a report on the computer, typing, or making calls to his clients, at these times, he will smoke without stopping. This may be a shortcoming for all males. The more busy or annoyed they are, the more they will smoke.” (FA08)
***Perceived benefits of or motivators for smoke-free homes***	
*Family’s health*	“First it is good for our health. Health is the most important point. Nothing else matters.” (FF04)
	“There will be less secondhand smoke and thirdhand smoke, and it can ensure family’s health.” (FA04)
*Children’s health*	“When I was pregnant and when child was still very little, I often discussed with my husband about whether to let people smoke in our home or not. Because we were afraid smoking is bad to his growth, I told my husband not to smoke at home….” (FA01)
*Role model for children*	“My children rarely see me smoking because I don’t want them to see the bad habit. In this way, there will be positive influence on children’s development. Especially to young boys, I don't want him to smoke when he grows up.” (MF01)
*Clean environment, no smell*	“Our home would be clean, without pollution. If someone smokes inside, cigarette ash will be everywhere. It is bad for children.” (FF03)
	“At least there will not be smoke smell, and the air will be fresh. That would be good for the health of the family.” (FA03)
	“First, the house will be cleaner, and smoke smell will not be so strong…. Rooms where people smoke, like our office, the walls have turned yellow.” (MF02)
*Avoiding embarrassment*	“For example, guests want to smoke. If you ask them not to smoke on the spot, it must be embarrassing. If you tell them clearly at the beginning that your home is smoke-free, then these embarrassments could be avoided. In addition, it ensures the health of older adults and children.” (MA02)
*Potential impact on smoking behavior*	“Because it only asks the person to smoke outside of the house, but not force the person to quit smoking. [They] will still continue to smoke. Outside of the door, in the hallway, or going outside, smoking still continues.” (MA03)
	“I don’t think it is helpful. He smokes in the office and I can’t control him when he is not at home. We do not work in the same place.” (FF04)
	“Despite the fact that smokers still smoke outside, the amount of their smoking will definitely decrease. For example, when I go to a friend’ home where smoking is only allowed on the balcony, I will feel embarrassed if I go outside for smoking frequently. Therefore I can control the amount I smoke.” (MA01)
	“When you keep watching them and pushing them, they will have some impression in their brain. It is sort of helpful.” (FF06)
***Initiation of discussions about whether to allow smoking in the home***	
*Establish the rule early in the relationship*	“Of course, I brought it up first. This should be settled when you are getting married. We reached an agreement when we got married. I was against smoking, but I still tolerate it.” (FF02)
	“My wife often talks of this issue with me. We made the rule at the beginning that I am not allowed to smoke anywhere in our home.” (MA03)
	“I established them at the beginning. My wife keeps an eye on it. We work with each other pretty well.” (MA01)
*No discussion*	“We cannot discuss it. Discussing this is like gunpowder exploding. I can only hope that these reports become more frequent to influence him subconsciously. He is a stubborn person, and we cannot communicate about this problem. It is impossible to have a smoke-free home, so I no longer mention it.” (FA08)
***Decision-making authority regarding home smoking rules***	
*Wives*	“Yes. I have more power to say in regard to this point.” (FF04)
	“Of course, my wife does [have the authority].” (MA05)
*Children*	“My daughter. She comes back from school in the evening and often asks me not to smoke. She said smoking is harmful to health and even wanted to stick a non-smoking sign on my face.” (MA06)
*Shared authority*	“We all talk about that. My daughter also opposes smoking at home. [When she smells the smoke on his father, she will ask her father not to smoke.] There is not much difference regarding who has the most saying.” (FF06)
*Extended family*	“Yes, my mother and I have the power to make decisions. Non-smoking people have the right to speak.” (FF01)
***Husband’s response to conversations about smoke-free home policy***	
*Ignoring the request*	“He can't help smoking. I think it makes no difference whether I talk with him about this or not because he is a heavy smoker. Generally he would not smoke in son’s presence.” (FA05)
*Not smoking at home for a few days but then starting again*	“There were some quarrels when I oppose him smoking. After that, he might not smoke at home for a period of time, but a few days later he would smoke again. So that's why there are often some conflicts and fights.” (FA04)
*Insist on smoking anyway*	“Anyway at home, I will smoke when I want. The rule depends on self-awareness. If my son is doing homework at home or something else, I will smoke less. But even with the concern of children, I will not make my home smoke-free unless I am sick and have to lie in the bed and can’t smoke cigarette anymore.” (MA07)
*Devaluing the benefit of smoke-free homes*	“Unless someone has health problems or suffers from some diseases, it is impossible to establish the rule.” (FA05)
	“But frankly speaking, it means little to China, because the toxicity of the cigarettes must be smaller than the toxicity of powder dust. It is less harmful than the PM2.5 particles.” (MA02)
***Challenges and exceptions in enforcing smoke-free policies or rules***	
*Weather*	“You can’t ask him to smoke downstairs and outside every time when he wants to smoke. It is so cold and he has to change the clothes to go outside.” (FA07)
	“In the winter and at night, I think it is sort of cruel to ask them to go outside.” (FF09)
	“Like in a raining day or during the winter. People don’t want to go outside to smoke when the weather is bad.” (MA06)
	“But he may feel a bit wronged and sometimes he complains. For example, when the weather is cold, and he does not want to smoke on the balcony.” (FF01)
*Social gatherings, guests visiting*	“It depends on the person. It varies from person to person. Some people you ask him to go outside, and they are pretty self-aware [to comply]. Some people no matter what you say, they are still uncaring. Also, generally, when a group of people are gathering together, if you ask someone to go outside to smoke, it will make the person feel shamed or embarrassed.” (MF05)
	“When some guests who have close relationship with us come to visit. Because they didn't often come to my home, I felt embarrassed to ask them not to smoke. I would just let them smoke, and open the window to keep the room ventilated.” (FF05)
	“In traditional Chinese culture, guests are always respected. I will give them cigarettes. During those time the smoke smell is the strongest in our home.” (MF05)
*Family relationships*	“I am afraid that this regulation will trigger many family arguments and conflicts. I think this regulation doesn’t respect human rights.” (MA08)
	“I don’t know about other families, but I think that banning smoking inside the home will increase family tensions or stress.” (FA03)
	“My daddy is a smoker. When he comes I will open the windows and prepare a big astray with some water inside. After he smokes one cigarette, I will wash the astray so he can’t smoke any more unless he goes to outside stairs to smoke.” (FF03)
*Smoker at home alone*	“My husband could smoke secretly in the restroom. But basically I don’t allow him to smoke at home. When I go to sleep at night, I don’t know whether he smokes or not.” (FF05)
*Closing the door or opening the window to reduce the impact*	“For example, when it's very cold and raining heavily, smokers may smoke secretly when they go to restroom, or smoke in the kitchen. Although he shuts the door, the smell still spread out to the living room and bedrooms, which sometimes makes us argue again.” (FF02)
	“I smoke not necessarily because I want to ponder over things. It is very casual. I smoke toward the window just because the smoke can disperse quickly.” (MA08)
	“He will ask our daughter to leave the study room if he wants to smoke, and he would open the window even it’s cold outside. I think this is good enough.” (FA07)
*Less motivated with older children*	“And after our baby was born, as a father, he realized that smoking was bad for our baby even if I didn't emphasize it. Especially when the kid was young, he is more likely to make it. He would not smoke around the child as possible as he can. He would try to only smoke when taking the garbage out. Thus, from the time we got married and had our child, as a farther, he was gradually aware of his responsibility. However, as the child grows up, sometimes he is being lazy on this thing and secretly smokes a little bit.” (FF02)
*Addiction*	“I have thought about that but it seems not practical. My husband is so addicted to smoking. I have talked to him for many times but there is no solution.” (FA05)
	“My smoking addiction is quite strong, about two packs a day. Sometimes at home, even in the bedroom I will smoke. For example, if in the winter, the door and windows are open. I smoke in the bedroom, and other people will think that the smell is really bad, and got stung by the smoke badly. They keep complaining to me. But once my smoking addiction comes, I will still smoke.” (MA07)

Note: FA = Female nonsmoker without a complete smoke-free policy; FF = Female nonsmoker with a complete; MA = Male smoker without a complete smoke-free policy; MF = Male smoker with a complete policy.

### Description of smoking practices and rules in the home

When asked about smoking in the home, participants, specifically those with partial or no smoke-free policy, recollected locations where smoking most frequently occurred, triggers for smoking, or situations in which smoking occurred. Smoking was reportedly allowed in the living room most commonly (about half of those without a complete smoke-free policy), with a few mentioning the bathroom, kitchen, or bedroom. For roughly a third of those without a complete smoke-free home policy, the presence of a fan seemed to influence where smoking was allowed in some households. Smoking was prohibited in the bedrooms or around children for the vast majority of participants. When asked to describe triggers for smoking or situations in which smoking most commonly occurred, dinner or after dinner were mentioned by roughly half of participants without complete smoke-free homes. Using the computer, watching TV, playing mahjong, working at home, pondering problems, and using smoking to reduce worry and anxiety were each mentioned by at least one participant.

### Perceived benefits of and motivators for smoke-free homes

Participants who had smoke-free home policies were asked about the perceived benefits of a smoke-free home or why they implemented them. Those without a complete smoke-free policy were asked about the perceived benefits and why they might implement them. The themes were similar. The strongest theme was for the family’s health, and children’s health was a second strong theme, both of which were reported among the vast majority of participants. Other significant themes included: being a role model for children, cleaner environment and no smell, avoiding embarrassment among guests by being explicit about the rule, and the potential impact on smoking behavior, reported among both men and women. In terms of the latter reason, there were mixed responses, with some individuals believing it would have a positive impact and others believing that it would have no impact.

### Initiation of discussions about whether to allow smoking in the home

Among those households that had discussed whether to allow smoking in the home, the wife was credited with initiating the conversation in most circumstances. For a few families, the smoke-free policy started early in their marriage and was just accepted as natural. The husband initiated the conversation in just a few of the households. About a third of households without a complete smoke-free home had not discussed a rule to limit smoking in the home.

### Decision-making authority regarding home smoking rules

Participants also reported who most commonly had the authority to establish a smoke-free home. The majority of participants reported that wives had the most authority to do so; children also had a significant impact. In the majority of households, the authority was shared among all members of the family. The extended family was also mentioned as playing a role by a couple of participants.

### Husband’s response to conversations about smoke-free home policy

Participants were asked to describe how the husband of the dyad responded to conversations regarding a smoke-free home. When women were asked how their husbands responded to their requests to establish a smoke-free home, they indicated that their husbands agreed not to smoke at home or in front of family members, ignored the request, did not smoke at home for a few days and then started again, insisted on smoking in the home anyway, or devalued the benefits of having a smoke-free home. Husbands similarly reported that they would acquiesce to the request, at least for a short period, and a couple argued that smoking or secondhand smoke did not have a significant health risk.

### Challenges and exceptions in enforcing smoke-free policies or rules

When asked about potential difficulties in enforcing a home smoking rule, a range of themes emerged. The strongest was weather, with rain, winter, and cold temperature. Parties, social gatherings, and guests visiting were also viewed as difficult situations in which to enforce a smoke-free policy. Family visiting was noted frequently as difficult situations. Other notable challenges or exceptions included when the smoker was at home alone, closing doors or opening windows to circumvent rules, and being less motivated to avoid smoking in front of older children. Addiction also emerged as a strong barrier to enforcing a smoke-free policy.

## Discussion

This study was one of the first to qualitatively examine smoking practices in the home, perceived benefits of or motivators for establishing smoke-free homes, the process of establishing smoke-free home policies, and difficulties in enforcing or exceptions made in enforcing existing rules regarding smoking in the home in Shanghai, China. The most notable contributions to the literature were findings regarding how the rule was established; that is, what interpersonal processes occurred within household members leading to the adoption (or not the adoption) of smoke-free homes or smoking rules.

In regard to reasons for establishing a smoke-free home, the current findings are also consistent with prior research indicating that concern about the health impact of SHS was the most important reason for establishing a smoke-free home in Shanghai [10] and in other countries [13]. The other common reasons also resonate with prior quantitative findings indicating that child-related factors and cleanliness are important motivators [10,13]. In addition, there were mixed attitudes regarding the potential for promoting harm reduction or cessation as a result of implementing such a policy. Given some doubt among participants, this might be an important intervention message. In fact, studies have confirmed that a complete smoke-free home versus a home with some level of restrictions is more effective in increasing the likelihood of making quit attempts among the smokers [16]. Moreover, smokers who live in a smoke-free home have been shown to be more likely to have made a quitting attempt and maintain abstinence compared to smokers without a smoke-free home, indicating that smoke-free home policies act as a part of effective cessation support systems [16].

In terms of the interpersonal processes in the household resulting in the implementation of a smoke-free policy, women were seen as critical change agents. Despite prior research documenting that authoritative attitudes of husbands or father-in-laws were barriers to women influencing such policy adoption [9,11], the current study documented that women did have the authority to influence this matter, which was similarly found in research regarding the interpersonal processes that lead to the adoption of smoke-free homes in the U.S. [13]. Women in this study most commonly endorsed the idea and had the authority in many cases to make the decision to implement them. However, there were some situations where this was not the case and the discussion to implement a smoke-free policy was quite sensitive. Many participants who had a smoke-free policy had adopted it early on in their relationship. Thus, early relationship years or during the beginning of the marriage may be a critical intervention window for addressing smoke-free homes. This also coincides with an important opportunity prior to beginning the family in which men might be particularly invested in the health of their home and family. The one child family planning policy in China promotes the value and status of children at home, which is a good opportunity to advocate for having a smoke-free home policy in families with children. Moreover, studies suggested that the lack of smoke-free home policies, even in homes without smoking parents, may weaken communication of parental antismoking values, while implementing smoke-free homes (despite the smoking status of the parents) might dissuade youth smoking [17]. This is in line with other research documenting the importance of parental attitudes toward smoking rather than their actual smoking behavior in terms of the impact on youth smoking initiation [18]. Furthermore, studies have confirmed that a complete smoke-free home versus a home with some level of restrictions is more effective in reducing the likelihood of adolescents smoking [16]. These findings and prior research might help address the interpersonal issues related to persuading smokers to commit to a smoke-free policy rather than ignore the request or lapse into smoking in the home after agreeing to the policy, which were prominent themes of husbands’ reactions to the policy in the current study and in prior research [13].

Our findings indicated several issues with enforcement. In terms of where smoking was most commonly allowed, our findings were similar to prior quantitative findings indicating that, among participants with no or partial smoke-free policies, the most common places where smoking was allowed included the living room, kitchen, and bathroom [10]. Moreover, there were enforcement challenges in terms of several harm reduction behaviors, such as smoking near windows or by fans in order to reduce the impact of SHS exposure. Prior research has also found that the misconception regarding these harm reduction behaviors is prevalent in China [19]. Guests visiting, social gatherings, and family relationships also were reported as particular challenges in enforcement, which is highly related to the cultural context of smoking in China. That is, prior research has highlighted the identification of smoking as a symbol of personal freedom, the importance of tobacco in social and cultural interactions, and the importance of tobacco to the economy in China [19].

It was also interesting to note that, as children aged, some smokers were less vigilant about protecting them from SHS. In fact, prior research has found that having children under 18 years old was not associated with having a complete smoke-free home policy, which is consistent with other studies [10,16,17]. People tend to believe that older children may not be sensitive to SHS. Educational outreach should grasp these opportunities and focus on the information that SHS is dangerous to all nonsmokers of all ages, including older children and adolescents.

Finally, the impact of addiction on the feasibility of establishing and enforcing a smoke-free home policy in this context was important to note. The wave 3 International Tobacco Control survey in China conducted in 2009 found that 96% of adult smokers were daily smokers, and adult male daily smokers in China smoked an average of 17 cigarettes per day [20]. Thus, implementing a smoke-free home policy in this context might be challenging, particularly if homes do not have highly accessible outdoor areas where smokers can go to smoke.

This study has important implications for research and practice. Research is needed to refine measures regarding interpersonal interactions that facilitate or impede the adoption of smoke-free home policies. Moreover, intervention strategies targeting the motivators for implementing smoke-free policies to promote their adoption (e.g., health of family and children) and targeting the challenges in adopting and enforcing them (e.g., weather, concerns during social gatherings, less concern about the impact of SHS on older children) should be developed and tested to identify ways to reduce SHS exposure among youth in China. In particular, it may be beneficial to address the interpersonal processes to aid women and children in their communication with smokers in the home regarding SHS exposure and smoke-free homes in order to maximize the effectiveness of these opportunities. In practice, clinicians should promote smoke-free homes in the practice setting, particularly among youth who have specific vulnerabilities that could be exacerbated by SHS such as asthma. The school setting may also be a place where children can be educated about the harms of SHS exposure and may be empowered as change agents in their home. As in other countries, prevalence of smoke-free homes is also likely to rise as smoke-free public places become more common [21].

### Limitations

This study has some limitations. First, this was a qualitative study of 30 male smokers and female nonsmokers with young children in Shanghai, China. Thus, findings from this small sample may not generalize to other adults in Shanghai or China more broadly. In addition, the interviews may not have yielded exhaustive information regarding the constructs and processes investigated; thus, additional qualitative and quantitative research is need to confirm and elaborate on these findings.

## Conclusions

The current study documented motivators for and perceived benefits of implementing smoke-free home policies, the interpersonal processes by which they are adopted, and enforcement challenges and exceptions. Particularly important findings focused on the important role of women in the process of adopting smoke-free home policies. The current findings suggest that specific factors, particularly the health of the family, could be highlighted to increase the importance of adopting a smoke-free home, that the process of adoption could be facilitated by assisting women, men, and children in navigating their communication, and that enforcement challenges must be considered in interventions.

## Abbreviations

SHS: Secondhand smoke
MPH: Masters of public health
SD: Standard deviation
